# Comparative assessment of the physical structure of antler and bovine bone substitutes: An in vitro study

**DOI:** 10.34172/japid.2024.012

**Published:** 2024-05-28

**Authors:** Mohammad Hossein Mahboubian, Mahdi Kadkhodazadeh, Reza Amid, Anahita Moscowchi

**Affiliations:** ^1^Dental Research Center, Research Institute for Dental Sciences, School of Dentistry, Shahid Beheshti University of Medical Sciences, Tehran, Iran; ^2^Department of Periodontics, School of Dentistry, Shahid Beheshti University of Medical Sciences, Tehran, Iran

**Keywords:** Biocompatible materials, Bone substitutes, Physical phenomena

## Abstract

**Background.:**

The use of bone graft materials has significantly increased. Given the inherent variations in structure and functionality between different grafting materials, this evaluated and compared the physical attributes of antler and bovine femur bone substitutes.

**Methods.:**

In the present in vitro investigation, the surface morphological architecture of the two bone substitutes with different origins was assessed through scanning electron microscopy. Furthermore, the Brunauer–Emmett–Teller (BET) technique was employed to measure the porosity, specific surface area (SSA), and pore morphology.

**Results.:**

Scanning electron microscopy observations indicated that the surface of the bovine particles appeared smoother, while the antler particles exhibited a rougher surface texture. The BET analysis revealed that both samples exhibited identical pore morphology. The SSA was 15.974 m^2^/g in the antler particles compared with 18.404 m^2^/g in the bovine sample. The total porosity volume in the antler and bovine femur bone substitutes were 0.2172 cm^3^/g and 0.2918 cm^3^/g, respectively. Additionally, the antler particles had a porosity percentage of 40%, whereas the bovine femur bone substitute showed a porosity percentage of 43.5%.

**Conclusion.:**

Based on the results of this study, it seems that the two samples of bone grafting materials have comparable physical structures.

## Introduction

 The reconstruction of bone defects resulting from trauma, inflammatory diseases, and tumors holds significant importance^1^ due to the potential consequences of leaving these lesions untreated, which may result in the formation of connective tissue.^2^ Consequently, addressing bone defects is crucial to any clinical procedure.^3^

 Despite having defined the ideal characteristics for a bone graft material over three decades ago, no material has been able to encompass all of these characteristics to date.^4^ The use of autogenous bone is widely regarded as the gold standard.^5^ In addition to proper histocompatibility, autografts do not elicit the immune response and possess essential properties for successful regeneration, including osteogenesis, osteoinduction, and osteoconduction.^6^ However, the use of autogenous bone presents challenges such as prolonged surgical time, potential complications in the donor site, and postoperative pain and discomfort. In addition, the potential for infection transmission and immune system stimulation associated with allografts has prompted the exploration of alternative biomaterials, such as xenografts and synthetic materials, to treat bone defects.^6-8^

 Numerous studies have demonstrated that using biological sources to prepare hydroxyapatite is a valuable approach for producing inexpensive and effective xenografts for bone regeneration.^9^ The key advantage of xenografts is their unrestricted availability. In addition, they possess biocompatibility, a porous structure, reasonable production costs, and mechanical strength.^10^

 Xenografts can be obtained from different species,^11^ with commonly available commercial xenograft products typically sourced from bovine origins that may raise ethical concerns due to the sacrifice of involved animals.^12^ The antler, unique to mammals, is the sole body part capable of complete regeneration and exhibits a remarkable growth rate of 2 to 4 cm per day.^13^ The physical and structural characteristics of antlers have garnered significant interest.^14^

 The biological response to transplant materials may be influenced by their physical attributes, including porosity, particle size, and shape.^15^ Therefore, this study aimed to investigate and compare the physical and structural characteristics of two bone substitutes derived from antler and bovine femur sources.

## Methods

 This in vitro study was carried out using particles with bovine femur bone origin (Bone^+^ B^®^, Novateb Pars Co., Iran) and a bone substitute derived from *Cervus elaphus* Maral’s antler (Maral Pajoohesh Shams Co., Iran).

 The morphological characteristics were assessed using a scanning electron microscope (SEM) with an operating voltage of 25.0 kV (TESCAN VEGA 3, TSCAN, Brno, Czech Republic). Porosity and specific surface area (SSA) were determined through Brunauer-Emmett-Teller (BET) analysis using 0.5 grams of each bone material. The bone substitutes were pretreated and degassed by vacuuming for 2 hours (FlowPrep 060, Micromeritics, GA, USA), with liquid nitrogen as the adsorptive. The saturated vapor pressure was set at 88 kilo Pascal, and the temperature was set at 77 K (TriStar II Plus, Micromeritics, GA, USA). SSA was expressed as square meters per gram of mineral (m^2^/g). The shape of the pores was determined through an adsorption-desorption diagram, while the porosity volume and mean pore diameter were measured using the BET equation. Both assays were conducted and reported by an individual blinded to the characteristics of the materials.

## Results

**Figure 1:**
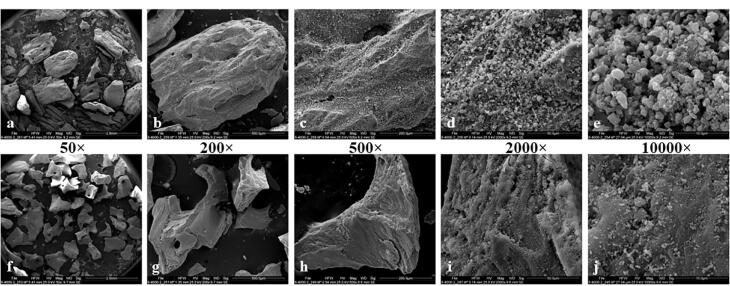
Surface morphology of bone substitutes: Antler (a-e) and Bovine (f-j). Magnification: a and f ( × 50), b and g ( × 200), c and h ( × 500), d and h ( × 2,000), e and j ( × 10000)

**Figure 2:**
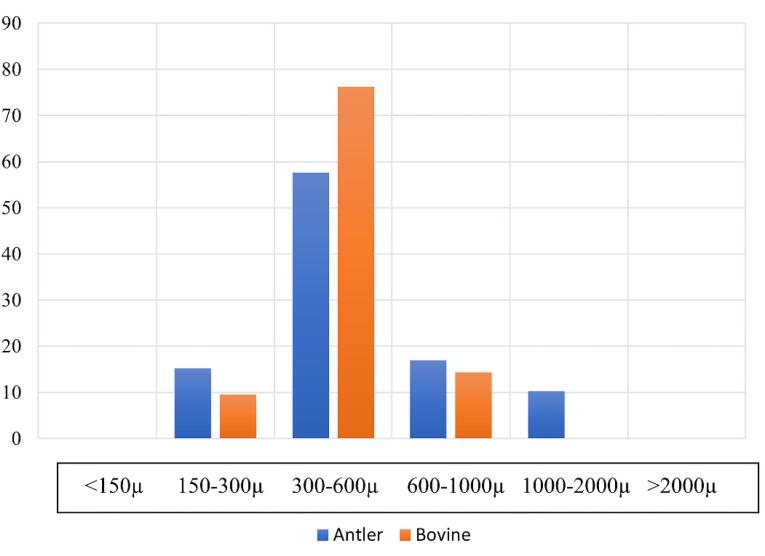
Particle size distribution of the bone substitutes

 The morphological analysis revealed that the antler-derived bone substitute exhibited more rounded angles than the bovine particles. The bovine grafting material particles displayed sharper and more fragmented edges (Figure 1). The particle size of the antler particles encompassed a wider range than the material derived from bovine femur bone (Figure 2). Furthermore, greater variation in the shape and size of particles was observed in the antler particles. The surface of the particles derived from bovine femur bone was smoother, while the antler particles exhibited more surface roughness. Both materials displayed holes ranging approximately 50‒300 µm in size. Layered structures were observed in both samples, with the grafting material derived from bovine femur bone exhibiting more pronounced visibility. Small nodules were present in both samples, but the antler sample exhibited a significantly higher density and larger size of these nodules. The majority of particles in both samples fell within a range of 300‒600 µm, with no particles smaller than 150 µm in either sample. Only the sample derived from antler origin contained particles with dimensions ranging from 1000 to 2000 µm.

**Figure 3:**
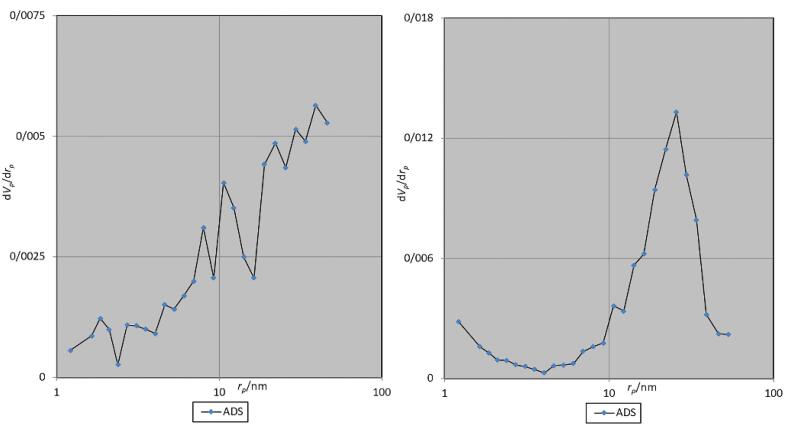
BJH diagram. Antler (left) and bovine (right)

 The pore morphology in both bone substitutes exhibited a slit-like structure. Table 1 presents the results of the BET analysis. The distribution of pores by diameter is depicted in the Barrett-Joyner-Halenda (BJH) diagram (Figure 3).

**Table 1:** Porosity and specific surface area

**Type of bone substitute**	**Porosity (%)**	**Density (g/cm**^3^**)**	**Specific surface area (m^2^/g)**	**Total porosity volume (cm**^3^**/g)**	**Mean hole diameter (nm)**
Antler	040	0.55	15.974	0.2172	54.377
Bovine	43.5	0.67	18.404	0.2918	63.41

## Discussion

 SEM revealed that the antler particles had more diverse sizes, which might be attributed to differences in their respective preparation methods. The majority of large particles in both samples exhibited holes ranging in size from 50 to 300 µm, which is consistent with the particle size of other xenografts. It has been demonstrated that cavities larger than 100 µm are crucial for the formation of blood vessels.^16^ A wide range of particle sizes might impede the angiogenesis process, as smaller particles tend to fill the interstitial spaces between larger particles and slow down this process.^17^ Furthermore, the antler particles had rougher surfaces. Previous research has demonstrated that smaller particles and a rougher surface of grafting materials elicit a stronger immune response, characterized by increased production of TNF-a and IL-6.^18^ This immune response is associated with the recruitment of cells to the regeneration site and the replacement of the graft material with new bone. Additionally, a rougher surface promotes better adhesion of osteoblasts, a stronger connection between the host bone and the graft material, and improved bone regeneration.^19^ The expression of osteoprotegerin, a receptor related to osteoclastogenesis, is likely to be higher in the antler particles due to surface roughness.^20,21^ The bovine particles had sharper angles, which might potentially result in Schneiderian membrane perforation during sinus augmentation procedures.

 Porosity in grafting substitutes allows the infiltration of cells into the material. The presence of these pores helps nourish and dispose of osteoblasts’ waste materials. The minimum size of porosity in bone graft materials is typically around 100 µm. However, the optimal size for porosity among materials is > 300 µm to ensure proper blood supply.^22-24^ Another crucial factor to consider is the surface characteristics of the graft material, as they significantly influence angiogenesis, the interconnection of bone cells, and their migration and proliferation.^4^ Furthermore, the impact of the origin of xenograft on its properties has been demonstrated.^25^

 Different techniques are employed to assess the porosity and specific surface of materials. The measurement of porosity with a size range of 1‒100 µm is accomplished using the mercury-assisted porosity measurement. On the other hand, the BET procedure is a precise method for evaluating pores within a range of 1‒100 nm. Alternatively, low-angle x-ray and neutron scattering approaches can be used to evaluate pores ranging from 0.4 to 2 nm.^26^ Given the necessity to examine the porosity of the samples at dimensions below 100 nm, the gas absorption method and BET theory were employed in the present study.

 The antler sample exhibited a lower percentage of porosity (40%) compared to the bovine bone substitute (43.5%). Notably, both materials demonstrated a lower porosity percentage than Bio-Oss (70.5%).^25^ In Zhang’s investigation, the porosity of the prepared xenograft from deer antler was 75%, which surpasses the porosity of the test material in this study.^27^ It is important to acknowledge that the animal’s preparation procedure, breed, and age may influence the porosity level. Furthermore, it is imperative to consider that the testing protocol might impact the outcomes.

 The BET analysis revealed that the bovine bone substitute micropores were, on average, 16.6% larger than those of the antler particles. These micropores in both materials are slightly larger and comparable to that of Bio-Oss (30 nm).^28^ Examining the pore size distribution, the antler sample exhibited greater dispersion, with a higher percentage of pores under 10 nm. The SSA in both samples was much higher than Puros (2 m^2^/g) and Creos allografts (0.025 m^2^/g).^29^ The sample obtained from bovine bone demonstrated a 34% increase in pore volume and a SSA 15.2% greater per gram compared to the antler sample. Consequently, due to its greater SSA, higher porosity percentage, and wider pore size, it appears that the bovine sample will undergo resorption at a faster rate. Bone graft materials that exhibit slower degradation are better suited for alveolar crest reconstruction, albeit necessitating a longer duration for repair.^30^

 The pores in both samples exhibited a continuous transverse extension within a single particle, displaying a slit-like configuration. This particular pore structure has been demonstrated to facilitate enhanced molecular and fluid mobility. Consequently, it appears that the morphology of the pores in both materials may contribute to intercellular signaling.^31^

 Further research should be conducted to compare this particular bone substitute with other grafting materials currently available on the market or other bone materials with different processing procedures. In addition, it is recommended to assess and compare the impact of the physical attributes of these two bone substitutes in animal studies, specifically in terms of osteopromotion levels, material absorption rates, replacement with new bone, and angiogenesis.

## Conclusion

 Based on the results of this study, it seems that the two samples of bone substitute originating from antler and bovine femur bone have similar physical structures. Both materials’ porosity, SSA, and density were almost identical. The bovine material displayed a limited range of particle sizes. On the other hand, the antler bone substitute particles exhibited a rougher surface texture that might enhance osteoblast adhesion.

## Consent for Publication

 Not applicable.

## Competing Interests

 The authors do not have any financial interest in the companies whose materials were included in this study.

## Data Availability Statement

 The data will be shared upon reasonable request by the corresponding author.

## Ethical Approval

 This study was approved by the Ethics Committee of Shahid Beheshti University of Medical Sciences, Tehran, Iran (IR.SBMU.DRC.REC.1401.002).

## Funding

 The study was funded by Shahid Beheshti University of Medical Sciences.
